# Difference in Optical Coherence Tomography Angiography Parameters After SARS-CoV-2 Infection During the Alpha and Delta Variant Dominance Periods

**DOI:** 10.3390/v17010047

**Published:** 2024-12-31

**Authors:** Magdalena Kal, Michał Brzdęk, Izabella Karska-Basta, Piotr Rzymski, Antonio Pinna, Jerzy Mackiewicz, Dominik Odrobina, Dorota Zarębska-Michaluk, Robert Flisiak

**Affiliations:** 1 Collegium Medicum, Jan Kochanowski University, 25-317 Kielce, Poland; 2 Ophthalmic Clinic, The Voivodeship Hospital, 25-736 Kielce, Poland; 3 Department of Gastroenterology, Medical University of Lodz, 92-213 Lodz, Poland; 4 Department of Ophthalmology, Faculty of Medicine, Jagiellonian University Medical College, 31-501 Krakow, Poland; 5 Clinic of Ophthalmology and Ocular Oncology, University Hospital, 31-501 Krakow, Poland; 6 Department of Environmental Medicine, Poznan’ University of Medical Sciences, 60-806 Poznan, Poland; rzymskipiotr@ump.edu.pl; 7 Department of Medicine, Surgery, and Pharmacy, Ophthalmology Unit, University of Sassari, 07100 Sassari, Italy; apinna@uniss.it; 8 Department of Vitreoretinal Surgery, Medical University of Lublin, 20-079 Lublin, Poland; 9 Institute of Medical Science, Jan Kochanowski University, 25-317 Kielce, Poland; 10 Department of Infectious Diseases and Allergology, Jan Kochanowski University, 25-317 Kielce, Poland; 11 Department of Infectious Diseases and Hepatology, Medical University of Białystok, 15-540 Białystok, Poland; robert.flisiak1@gmail.com

**Keywords:** COVID-19, Alpha variant, Delta variant, vessel density, optical coherence tomography angiography, foveal avascular zone

## Abstract

The SARS-CoV-2 infection manifests with diverse clinical manifestations, with severity potentially influenced by the viral variant. COVID-19 has also been shown to impact ocular microcirculation in some patients, but whether this effect varies by viral lineage remains unclear. This prospective study compared clinical features and ocular parameters assessed via optical coherence tomography angiography (OCTA) in patients recovering from SARS-CoV-2 infections during the dominance of two distinctive viral lineages, Alpha (B.1.1.7) and Delta (B.1.617.2), and compared them to a control group. The following parameters were measured: vessel density (VD) in the superficial capillary plexus (SCP), deep capillary plexus (DCP), and choriocapillaris (CCP) using OCTA, with a manual assessment of the foveal avascular zones in the SCP (FAZs) and DCP (FAZd). A control group was also included. Among 63 patients in the Alpha group and 41 in the Delta group, no eye-related symptoms were reported during the examination. However, the Delta group showed significantly lower VD in the SCP and DCP across all quadrants (*p* < 0.001–0.039), while the Alpha group showed reduced VD in the foveal CCP (*p* = 0.005) and significantly wider FAZs and FAZd (*p* = 0.002 for both). In conclusion, ocular microcirculatory changes differed between the two variants, with Alpha associated with foveal choroidal VD reduction and larger FAZs and Delta linked to lower SCP and DCP VD across multiple regions. These findings highlight the potential for SARS-CoV-2 variants to differentially impact ocular vasculature, underscoring the need for variant-specific follow-up in COVID-19 patients.

## 1. Introduction

The severe acute respiratory syndrome coronavirus 2 (SARS-CoV-2), the causative agent of Coronavirus Disease 2019 (COVID-19), first identified in 2019 in Wuhan, China, caused a pandemic leading to millions of deaths [1]. Initially, the virus was believed to primarily target the respiratory system, with severe cases leading to acute respiratory distress syndrome. However, subsequent research revealed that SARS-CoV-2 has a wide array of effects, leading to vascular injury, coagulation abnormalities, and adverse effects on various organs, including the heart, kidney, liver, and lymph nodes [2,3,4,5].

SARS-CoV-2 achieves cellular entry by binding to the angiotensin-converting enzyme 2 receptor, which is highly expressed not only in the respiratory system but also in vascular endothelial cells throughout the human body [6]. Endothelial dysfunction in COVID-19 involves a complex interplay of mechanisms, including endothelial cell damage, disruption of lipid metabolism, secretion of adhesion molecules by endothelial cells, secretion of chemokines by vascular smooth muscle cells, and subsequent recruitment of inflammatory cells such as monocytes, neutrophils, lymphocytes, and mast cells to the arterial walls [7]. Proinflammatory cytokines, especially necrosis factor-alpha, interleukin-1, and interleukin-6, are critical in disrupting the junctions between endothelial cells. They enhance the expression of other cytokines and adhesion molecules, leading to increased vascular permeability and atherosclerosis progression [8].

The clinical course of SARS-CoV-2 infection is influenced by numerous factors, including older age, male gender, obesity, and pre-existing conditions such as cardiovascular, respiratory, or renal disease, cancer, and immunosuppressive treatment [9]. Additionally, the presence of different SARS-CoV-2 variants affects the course of COVID-19. To better monitor and understand the evolving nature of the virus, the World Health Organization (WHO) has categorized SARS-CoV-2 variants into three main groups: variants of interest, variants of concern, and variants under monitoring [10].

SARS-CoV-2 exhibits a relatively high rate of evolution. The first genome of the virus was sequenced on 10 January 2020 and, within just 13 months, 468,000 virus sequences were documented [11]. The Alpha variant (lineage B.1.1.7) was first detected in Kent, UK, and had high transmission and virulence. This variant spread to 189 countries and was no longer reported after September 2021 [12]. The Alpha variant predominated in Poland from March to June 2021 [13]. The Delta variant (lineage B.1.617.2) was first identified in India in October 2020. Observations showed that patients infected with the variant were more likely to be hospitalized compared to the Alpha variant. The Delta variant became the most dominant strain worldwide between March and July 2021 [14]. The WHO classified the Delta variant as a variant of concern on 11 May 2021 [15]. In Poland, the Delta variant predominates from August to December 2021 [16].

The retinal vasculature reflects the state of the systemic microvascular health. In COVID-19 patients, fundoscopic examinations have revealed small hemorrhages, ischemic areas such as cotton wool spots, and sectoral retinal ischemia. Optical coherence tomography–angiography (OCTA) is useful for noninvasive assessment of macular vessel density. Studies utilizing OCTA have demonstrated reduced vessel density and reduced inner retinal volume in COVID-19 patients, both during the acute phase of the disease and after recovery. These changes are particularly pronounced in critically ill patients, suggesting that impaired retinal microcirculation may be a long-term consequence of severe infection [17].

Swept-source optical coherence tomography (SS-OCT) is a cutting-edge advancement in retinal imaging that eliminates artifacts and produces histopathologically similar images of the retina and choroid. OCTA detects signals caused by moving red blood cells (RBCs) as a contrast mechanism to visualize blood flow in retinal, choroidal, and peripapillary vessels, offering precise insights into vascular alterations [18].

In the present study, we analyzed clinical data and ophthalmologic parameters, including OCTA parameters in adult patients hospitalized during the two pandemic periods (March to May 2021 and November to December 2021) corresponding to the predominance of Alpha and Delta variants, respectively. This study aims to expand our understanding of the variant-specific systemic and ocular effects of SARS-CoV-2, contributing to the broader field of post-viral microvascular research and highlighting the role of OCTA in assessing these changes.

## 2. Materials and Methods

### 2.1. Materials

A prospective ophthalmological study was performed among two COVID-19 groups who were infected during the dominance periods of different SARS-CoV-2 variants: Alpha and Delta. The Alpha group consisted of 63 patients (120 eyes) hospitalized at the Department of Infectious Diseases of the Voivodeship Hospital in Kielce, Poland, from March to May 2021. Patients were examined ophthalmologically 2–8 weeks after the end of their hospitalization. The Delta group included 41 patients (77 eyes) hospitalized in the same hospital department in November and December 2021.

In both the Alpha and Delta groups, SARS-CoV-2 infection was confirmed by polymerase chain reaction (PCR). Chest computed tomography (CT) confirmed pneumonia with typical lesions in 26 patients in the Alpha group and in 3 patients in the Delta group.

Diseases such as diabetes, stroke, myocardial infarction, and autoimmune diseases were excluded from both hospitalized groups. The following ophthalmic exclusion criteria were applied in both groups: myopia > −3 diopters, hyperopia > +3 diopters, diseases of the central and peripheral retina, diseases of the optic nerve, previous intraocular surgery, a history of uveitis, a history of eye trauma, and opaque optic centers of the eye affecting the quality of the OCT scan. Age criteria of 30 to 70 years were used in both groups.

In the Alpha group, an initial cohort of 94 patients (188 eyes) was examined after hospital discharge. The following patients were excluded: 10 patients with age-related macular degeneration (AMD), 13 with diabetes, 2 with glaucoma, 1 with hyperopia > +3.0 diopters, 3 with myopia > −3.0 diopters, and 2 after cataract surgery.

After applying these exclusion criteria, the final Alpha group comprised 63 patients (126 eyes). From this group, the following eyes were excluded: 2 eyes with high hyperopia higher than +3.0 Diopters, 1 eye with high myopia higher than −3.0 Diopters, 2 eyes after ocular trauma, and 1 eye treated for uveitis.

The Delta group initially included 44 patients (88 eyes) after hospital discharge and excluded the following: 1 person with glucose intolerance, 1 person with familial spastic paraplegia, and 1 person due to technically poorly performed OCTA scans. In the Delta group of 41 subjects (77 eyes), the following eyes were excluded: 1 after cataract surgery, 2 with hyperopia, and 1 with mature cataracts.

The control group included 43 (83 eyes) healthy patients who visited the ophthalmology department for a routine eye examination. The following inclusion criteria were used: age 30–70 years, negative laboratory tests for SARS-CoV-2 infection (PCR from nasopharyngeal swab), no history of COVID-19 history or close contact with patients with COVID-19 within 14 days before the study, and absence of concurrent ocular disease.

### 2.2. Optical Coherence Tomography Angiography Examination

All scans in the OCTA study were obtained with a Swept Source DRI-OCT Triton SS-OCT Angio (IMAGEnet61.34.19388, Topcon Inc., Tokyo, Japan). OCTA images were captured using 6 × 6 mm scanning protocols.

**Figure 1:**
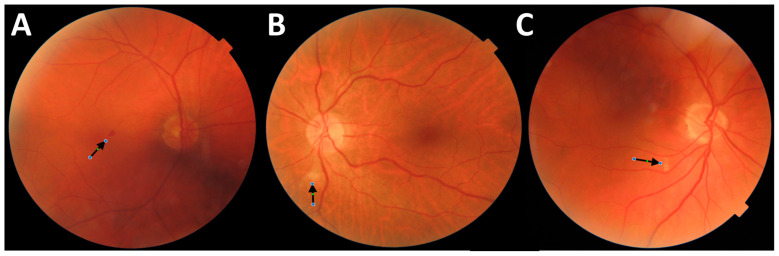
(**A**) The image of the fundus of the right eye in a COVID-19 patient from the group Delta. The arrow shows preretinal hemorrhage located in the paracentral part of the macula. (**B**) The image of the left eye fundus in a COVID-19 patient from the group Delta. The arrow shows a cotton wool spot located below the optic disc (**C**). The image of the right eye fundus in a COVID-19 patient from the group Delta. The arrow shows a cotton wool spot located in a paracentral part of the macula. All images were obtained by Triton’s fundus camera (author’s archive).

The Triton urinalysis allows for the automatic measurement of such OCTA parameters as vessel density (VD) in three different plexuses: superficial capillary plexus (SCP), deep capillary plexus (DCP), and choriocapillaris (CCP), using Early Treatment Diabetic Retinopathy Study (ETDRS) grid subfields to define areas of interest. VD is calculated in the following areas: foveal (F), superior (S), nasal (N), inferior (I), and temporal (T). The mean density (Mean VD) was calculated as the average value obtained in the parafoveal area, defined as the area conformed by the superior (S), nasal (N), inferior (I), and temporal (T) ETDRS subfields centered on the macula by fixation. Two independent evaluators manually calculated the area of the foveal avascular zone (FAZ) in the SCP (FAZs) and DCP (FAZd) encompassing the fovea, where no clear and demarcated vessels were visible in OCTA. Scans with an image quality of at least 65% were eligible for the study. Using Triton’s fundus camera, we visualized changes in the fundus of the eye in Delta group patients as the preretinal hemorrhage (Figure 1A) and cotton wool spot (Figure 1B,C).

### 2.3. Treatment of Patients

Patients with COVID-19 in both Alpha and Delta groups admitted to the hospital received treatment in accordance with the recommendations at that time upon hospital admission [19]. Remdesivir was administered intravenously at an initial dose of 200 mg on the first day and 100 mg for the following four days. Dexamethasone was administered at a daily dose of 4–8 mg orally or intravenously for 7–10 days. Tocilizumab was administered intravenously at a single dose of 400–800 mg, depending on the patient’s weight. Interleukin-6 levels were an indication for the administration of tocilizumab [20]. Low-molecular-weight heparin was administered prophylactically or in therapeutic doses in a weight-dependent dose according to the instructions on the label.

### 2.4. Statistical Analysis

Distributions of quantitative traits were checked. The means (Ms) and standard deviations (SDs), as well as medians (Mes) and interquartile ranges (IQRs), were calculated for them. Two tests, the Student’s *t*-test and the Mann–Whitney test, were used to determine differences between the means, which were applied according to the distributions of the variables. Correlations between saturation (a marker of tissue oxygenation), interleukin-6 (a marker of inflammation), and d-dimer (a marker of hemostatic abnormalities and intravascular thrombosis) and OCTA parameters were assessed with Spearman’s correlation coefficient (Rs). For qualitative characteristics, counts and percent frequencies were counted, and relationships between variables were determined using the non-parametric chi-square test. Values were statistically significant at *p* < 0.05.

### 2.5. Ethical Considerations

This study was approved by the Bioethics Committee of the Collegium Medicum of Jan Kochanowski University in Kielce, Poland (study code 54 was approved on 1 July 2021). Patients signed consent for ophthalmologic examination.

## 3. Results

### 3.1. Demographic, Ocular, and Systemic Characteristics

The Alpha group included 63 (120 eyes) patients [20 females and 43 males (68.3%)] with a mean age ± SD of 51.3 ± 11.5 years, and the Delta group included 41 (77 eyes) patients with a mean age ± SD of 53.7 ± 11.9 years (Table 1). No statistically significant differences were found between the two groups regarding age or gender. Patients in the Alpha group revealed a significantly higher body mass index in comparison to the Delta group (*p* = 0.002).

Patients in both groups with COVID-19 did not complain of any ocular symptoms during eye examination. The mean ± SD of axial length was 23.6 ± 0.9 mm in the Alpha group and 23.6 ± 0.9) mm in the Delta group (*p* = 0.921). The mean ± SD of LogMAR Visual acuity was 0.30 ± 0.0, and the mean LogMAR Reading Vision was 0.3 ± 0.0 in both groups. The mean ± SD of intraocular pressure (IOP) was 16.4 ± 2.6 mmHg in the Alpha group and 16.2 ± 3.0 mmHg in the Delta group (*p* = 0.553). Headache as a neurological symptom was reported in 14.3% (9/63) of patients in the Alpha group and 9.8% (4/41) of patients in the Delta group, with no statistically significant difference observed between the two groups.

Hypertension was in 20 subjects (31.8%) in the Alpha group and 14 subjects (31.2%) in the Delta group (*p* = 0.203), and dyslipidemia was in 3 patients (4.8%) in the Alpha group and 7 patients (17.1%) in the Delta group (*p* = 0.037). Ischemic heart disease was in two patients in the Alpha group (3.8%) but in three patients in the Delta group (*p* = 0.350).

The control group consisted of 43 patients (*n* = 43; eyes = 83) with a mean ± SD age of 47.8 ± 1.4. All patients had a mean distance vision of 0.0 on the LogMAR scale and a mean ± SD reading vision of 0.3 ± 0.0 on the LogMAR scale; the mean ± SD spherical equivalent was 0.7 ± 0.1, and the mean ± SD axial length was 23.4 ± 0.1 mm.

The Alpha group had a significantly higher body mass, BMI, and spherical equivalent compared to the control group, whereas the Delta group exhibited a significantly higher age and spherical equivalent than the control group (Table 2).

### 3.2. Comparison of Laboratory and Imaging Between Alpha and Delta COVID-19 Patients on Admission

Mean blood saturation at admission and inflammatory markers such as C-reactive protein (CRP), procalcitonin, and interleukin-6, as well as blood cell counts (white blood cells, lymphocytes, neutrophils, and platelets), showed similar values between the groups, with *p*-values indicating no significant differences (Table 3). The only notable difference was in D-dimer levels, which were significantly higher in the Delta group (*p* = 0.005). Hyperinflammatory reaction, defined as the presence of IL-6 > 100 pg/mL, CRP > 100 mg/L, and D-dimer > 500 ng/mL, was reported in two patients in the Alpha group (3.2%) and one patient in the Delta group (2.4%), with no statistically significant difference. We did not observe any distinct changes in vascular retinal patterns in these individuals. Lung CT scans revealed lesions typical of SARS-CoV-2 infection in 26 patients in the Alpha group compared to only 3 patients in the Delta group (*p* = 0.001).

### 3.3. Comparison of Treatment Between Alpha and Delta COVID-19 Patients Admitted to Hospital

Oxygen supplementation was used in 23 patients in the Alpha group with a median (IQR) of 5 (6) days and, in the Delta group, in 21 patients for 6 (4) days. The need for continuous oxygen therapy did not differ significantly between the groups (*p* = 0.138), nor did the number of days requiring oxygen therapy (*p* = 0.879). Low-molecular-weight heparin was administered prophylactically to 59 Alpha group patients and Delta 38 patients in a weight-dependent dose (*p* = 0.847). Remdesivir was used in 26 patients in the Alpha group and 5 patients in the Delta group (*p* = 0.002). Hyperinflammatory syndrome was an indication for immunosuppressive drugs. Dexamethasone was administered in 22 patients in the Alpha group and in 14 patients in the Delta group (*p* = 0.935). Tocilizumab was administered to three patients in the Alpha group and in two patients in the Delta group (*p* = 0.978) (Table 4).

### 3.4. Comparison of OCTA Parameters Between Alpha and Delta COVID-19 Patients During Hospitalization

In the OCTA analysis, a significantly decreased mean ± SD of VD S SCP was observed in the Delta group compared to in the Alpha group (45.2 ± 2.63 vs. 48.3 ± 2.7, *p* < 0.001), as well as in VD N SCP (44.2 ± 2.7 vs. 45.1 ± 2.6, *p* = 0.039), VD T SCP (45.1 ± 2.1 vs. 46.7 ± 2.2, *p* < 0.001), and VD I SCP (44.8 ± 3.7 vs. 47.2 ± 4.4, *p* < 0.001). The mean VD SCP was significantly decreased in the Delta group compared to the Alpha group (44.8 ± 2.3 vs. 52.0 ± 2.4, *p* < 0.001). The mean ± SD of FAZs was significantly increased in the Alpha group compared to the Delta group (326.8 ± 107.1 vs. 282.1 ± 123.6, *p* = 0.002).

A significantly decreased mean ± SD of VD was observed in the DCP in the Delta group compared to the Alpha group in VD S DCP (47.3 ± 2.7 vs. 52.0 ± 3.3, *p* < 0.001), VD N DCP (45.4 ± 2.1 vs. 49.0 ± 3.0, *p* < 0.001), VD T DCP (43.5 ± 2.2 vs. 47.6 ± 2.5, *p* < 0.001), and in I VD DCP (44.5 ± 3.7 vs. 51.3 ± 4.0, *p* < 0.001). The mean VD DCP was significantly decreased in the Delta group compared to the Alpha group (45.2 ± 1.6 vs. 54.3 ± 2.6, *p* < 0.001). The mean ± SD of FAZd was significantly increased in the Alpha group compared to the Delta group (357.8 ± 144.3 vs. 270.0 ± 105.9, *p* = 0.002).

A significantly decreased mean ± SD of VD F CCP was observed in the Alpha group compared to the Delta group (51.4 ± 4.2 vs. 53.1 ± 3.9, *p* = 0.005). A significantly decreased VD was documented in the Delta group compared to the Alpha group in VD S CCP (51.5 ± 2.1 vs. 54.1 ± 2.0, *p* < 0.001), VD N CCP (52.3 ± 2.0 vs. 53.6 ± 2.1, *p* < 0.001), VD T CCP (53.2 ± 1.9 vs. 54.0 ± 1.9, *p* = 0.002), and in VD I CCP (51.9 ± 2.4 vs. 54.3 ± 2.2, *p* < 0.001). Furthermore, the mean VD CCP was significantly decreased in the Delta group compared to the Alpha group (52.2 ± 1.0 vs. 66.9 ± 1.3, *p* < 0.001) (Table 5). None of the OCTA parameters in the Alpha and Delta groups were significantly correlated with saturation, IL-6, and d-dimer levels (Table 6).

### 3.5. Comparison of OCTA Parameters Between Alpha COVID-19 Patients and Control Group

In the OCTA analysis, a significantly decreased mean ± SD of VD F CCP was observed in the Alpha group compared to in the control group (51.4 ± 4.2 vs. 52.4 ± 4.0, *p* = 0.046). Furthermore, the mean ± SD of FAZs and FAZd was significantly increased in the Alpha group compared to the control group (Table 7).

### 3.6. Comparison of OCTA Parameters Between Delta COVID-19 Patients and Control Group

In the OCTA analysis, the Delta group showed significantly reduced VD compared to the control group in VD S SCP, VD N SCP, VD I SCP, and VD T SCP. In DCP, most VD measures were lower in the Delta group, except for VD T DCP, which was slightly higher (*p* < 0.001). Similarly, in the CCP, VD S CCP was increased (*p* < 0.001) while other regions showed mixed results. The mean VD for the SCP, DCP, and CCP generally differed significantly between groups (all *p* < 0.005). FAZs and FAZd were also significantly increased in the Delta group (*p* = 0.002 for both) (Table 8).

## 4. Discussion

This study provides a comparative analysis of retinal microvascular changes in patients recovering from COVID-19 during the dominance of the Alpha and Delta SARS-CoV-2 variants, utilizing OCTA. By examining VD and FAZ parameters, this study highlights the distinct vascular effects of these two variants on the retina. These findings contribute to a growing understanding of the systemic and localized vascular implications of COVID-19 and underscore the utility of OCTA in assessing post-infection complications. Previous studies have documented ocular fundus abnormalities in patients infected with SARS-CoV-2, including microhemorrhages, cotton wool spots, and vascular tortuosity in the retina [21,22]. Marinho et al. reported the presence of cotton wool spots and microhemorrhages in adult COVID-19 patients 11–33 days after symptom onset, even in those not requiring intensive care. On OCT, these patients exhibited hyper-reflective changes in the retinal ganglion cell layer and inner plexiform layer [23].

In our study, we similarly identified hemorrhages and cotton wool spots in the central retina of several patients infected with the Delta variant. However, these lesions were absent in the Alpha variant group. These findings are consistent with the hypothesis that vascular endothelial dysfunction in COVID-19, driven by hypoxia, inflammation, and a hypercoagulable state, leads to retinal microvascular abnormalities.

The use of swept-source OCT (SS-OCT), a cutting-edge imaging technology, allowed us to generate histology-like images of the retina and choroid with unparalleled precision. The tunable light source, operating at a wavelength of 1050 nm and a scanning speed of 370,000 A-scans per second, enabled us to visualize deep retinal and choroidal structures. Furthermore, OCT angiography (OCTA) provides detailed maps of retinal and choroidal blood flow by detecting erythrocyte movement, enhancing our ability to assess vascular alterations [24,25].

To our knowledge, this is the first study to compare OCTA parameters between patients infected with different SARS-CoV-2 variants. Previous studies have primarily compared OCTA parameters between COVID-19 patients and healthy controls [26,27,28,29]. Our earlier work demonstrated a statistically significant difference between the Alpha and Delta variant groups in the foveal avascular zone (FAZ) area size [30].

We observed that hypertension was more prevalent in the Alpha group than in the Delta group, though this difference was not statistically significant. Chronic hypertension is known to cause structural alterations in the vascular endothelium, shifting the autoregulatory plateau in cerebral and ocular circulation to higher pressures. This may result in a reduced ability to dilate blood vessels during periods of decreased perfusion pressure, increasing the risk of ischemia [23,31]. Similar mechanisms may affect retinal circulation, though the specific impacts of hypertension and atherosclerosis on the retina in COVID-19 patients remain underexplored [32,33].

Inflammatory markers such as CRP, procalcitonin, lymphocytes, neutrophils, and interleukin-6 were slightly elevated in the Alpha group, while D-dimer levels were significantly higher in the Delta group. These findings suggest that the Delta variant may induce more pronounced coagulation abnormalities, potentially contributing to the more extensive retinal vascular changes observed in this group. Despite these differences, both groups received comparable oxygen therapy, anticoagulation, and corticosteroid treatments. The Alpha group received remdesivir more frequently, which may reflect evolving treatment protocols during the pandemic.

The FAZ, supplied by the choriocapillaris, is highly sensitive to ischemia and hypoxia. Enlargements of the FAZ have been observed in vascular diseases such as diabetic retinopathy and retinal vein occlusion and are similarly expected in COVID-19 due to hypercoagulability, hypoxia, and inflammation [34,35]. In our study, the Alpha group exhibited a significantly larger FAZ area than the Delta group.

Additionally, we found a higher vessel density (VD) in the superficial and deep capillary plexuses (SCP and DCP) in the Alpha group compared to the Delta group. These findings suggest that the Delta variant may have a more pronounced impact on retinal microcirculation, potentially reflecting greater vascular dysfunction. Interestingly, in our earlier work comparing the Alpha variant group with healthy controls, only a decrease in VD in the choriocapillaris was statistically significant. In contrast, the Delta variant group demonstrated significant alterations across nearly all retinal regions compared to controls, further underscoring the variant-specific differences in microvascular impact.

The vascular endothelium plays a crucial role in regulating retinal blood flow and maintaining the integrity of the blood–retinal barrier. Endothelial dysfunction due to inflammation, hypoxia, and vasospasm can lead to ischemia and vascular remodeling [36]. Retinal blood flow is also dynamically regulated in response to oxygen levels: hypoxia induces vasodilation and increased flow while hyperoxia triggers vasoconstriction [37,38]. Studies have shown that these mechanisms operate differently in larger vessels than in capillaries, with capillaries as key sites of vascular resistance [39].

OCTA studies in healthy individuals have demonstrated that hyperoxia reduces VD in the DCP while hypoxia increases it. However, the SCP appears as less responsive to such changes, highlighting the distinct metabolic roles of retinal vascular layers [40,41]. These findings align with our observation of decreased VD in the central choroid and increased VD in the parafoveal choroid in the Alpha group compared to the Delta group. The lack of comparable studies examining choroidal circulation across COVID-19 variants underscores the novelty of our findings.

We observed a statistically significantly lower VD in the central part of the choroid and a statistically significantly higher VD in its parafoveal part in the Alpha group compared to the Delta group. However, we cannot directly compare our results to other works analyzing the results of the choroidal circulation according to a specific COVID-19 variant as there have been no such comparisons in the literature to date. Our earlier work compared this parameter between the Alpha and healthy groups. We found that foveal VD was statistically significantly lower in the Alpha group in choriocapillaris compared to healthy subjects. Still, in the parafoveal portion, there were no statistically significant differences between these two groups [30]. Gonzales-Zamora et al. found no statistically significant differences in VD CCP between COVID-19 patients and healthy patients but did not perform a comparative analysis between the different COVID-19 groups [26]. Despite the lack of statistically significant changes in saturation between the two COVID-19 groups analyzed in our study, it should be noted that a statistically significantly higher number of patients in the Alpha group had typical lung lesions on chest CT. This may suggest that the Alpha group was thus more vulnerable to hypoxia. The choroid is a highly vascularized structure in the body and provides nutrition to the optic nerve, the retinal pigment epithelium, and the outer retinal layers that house the photoreceptors. It is the only metabolic source in the FAZ area. Oxygen supply to the retina also depends on choroidal blood flow [42].

Our study has several limitations. Ophthalmological assessments in both Alpha and Delta groups were not carried out during hospitalization but afterward. This decision was influenced by logistical constraints and the need to minimize the risk of infection among medical staff. During hospitalization, the primary focus was on treating pneumonia and preventing thromboembolic complications, leaving ophthalmological examinations as a secondary consideration. The ophthalmologic examinations were conducted at different times during the recovery period, ranging from 2 to 8 weeks post-discharge. This variation in timing likely introduced some degree of variability in the assessment of OCTA parameters, as the recovery process following SARS-CoV-2 infection is known to vary significantly among individuals. Individual healing trajectories could have influenced the observed vascular changes. Moreover, patients who survived despite treatment in the intensive care unit were not examined ophthalmologically. Additionally, the potential effect of vaccination against SARS-CoV-2 on OCTA parameters was not analyzed, nor was the influence of specific treatment on these ophthalmic outcomes. Furthermore, only a single OCTA ocular examination was performed during the recovery period, limiting our ability to assess the progression or resolution of retinal microvascular changes over time. Lastly, while our findings suggest that the Alpha and Delta SARS-CoV-2 variants have distinct effects on retinal vasculature, we did not explore the structural differences or specific mechanisms underlying these effects. Despite these limitations, our study has notable strengths. The study groups were carefully selected from the two pandemic waves, Alpha and Delta, and patients were unencumbered by advanced age, diabetes, and history of thromboembolic incidents. Additionally, we included a control group, enhancing our findings’ reliability. To our knowledge, there have been no similar comparative analyses between the different SARS-CoV-2 variants.

## 5. Conclusions

This study reveals significant differences in ocular microvascular parameters between patients recovering from COVID-19 during the Alpha and Delta variant waves. The Alpha variant was associated with reduced vessel density (VD) in the foveal region of the choriocapillaris (F CCP) and wider FAZs and FAZd, suggesting a localized impact on the foveal microvasculature. In contrast, the Delta variant caused a more widespread reduction in VD across the superficial capillary plexus (SCP) and deep capillary plexus (DCP), indicating more extensive retinal vascular involvement. These findings underscore the varying degrees of retinal microvascular changes induced by different SARS-CoV-2 variants. While the Alpha variant appears to cause relatively localized alterations, the Delta variant exerts a broader and potentially more severe impact on the retinal microcirculation. This highlights the utility of OCTA as a sensitive tool for detecting subtle post-COVID-19 vascular abnormalities, offering insights into the systemic effects of SARS-CoV-2 infection. Nevertheless, the long-term implications of these microvascular changes for retinal health and ocular function remain unclear. It is imperative to investigate whether these vascular alterations persist or resolve over time and to evaluate their potential impact on visual acuity and overall ocular health. Future research should also explore whether certain patient subgroups—such as those with comorbidities or more severe COVID-19 symptoms—are at higher risk of persistent retinal changes. Additionally, examining the effects of vaccination status and specific COVID-19 treatments on retinal outcomes could provide valuable insights into mitigating these complications.

## Figures and Tables

**Table 1:** Demographic, ocular, and systemic characteristics of two COVID-19 study groups, Alpha and Delta.

Variables	COVID-19 Patients Alpha Group		COVID-19 Patients Delta Group		*p*
	Mean (SD)	Median (IQR)	Mean (SD)	Median (IQR)	
Men, *n* (%)	43 (68.3) ^1^		17 (58.5) ^1^		0.312 ^2^
Women, *n* (%)	20 (31.7) ^1^		24 (41.5) ^1^		
Age (years)	51.3 (11.5)	51.0 (18.0)	53.71 (11.9)	54.0 (14.0)	0.313 ^3^
Body height (cm)	173.1 (9.5)	176.0 (15.0)	170.5 (8.6)	172.0 (14.0)	0.156 ^3^
Body mass (kg)	85.4 (15.6)	88.0 (20.0)	77.5 (19.8)	75.0 (16.0)	0.002 ^4^
BMI (kg/m^2^)	28.4 (4.1)	28.0 (6.0)	26.5 (5.4)	25.28 (3.5)	<0.001 ^4^
LogMar Visual acuity	0.3 (0.04)	0.3 (0.0)	0.3 (0.0)	0.30 (0.0)	0.767 ^4^
LogMar Reading vision	0.00 (0.02)	0.5 (0.0)	0.0 (0.0)	0.00 (0.0)	0.844 ^4^
Intraocular pressure mmHg	16.4 (2.6)	16.5 (3.8)	16.2 (3.0)	16.34 (5.4)	0.553 ^3^
Spherical equivalent (D)	0.13 (1.5)	0.0 (2.3)	−0.06 (1.4)	0.0 (1.5)	0.536 ^4^
Axial length (mm)	23.6 (0.9)	23.5 (1.1)	23.6 (0.9)	23.5 (1.0)	0.921 ^4^
Hypertension, *n* (%)	20 (31.8) ^1^		14 (31.2)		0.203 ^2^
Dyslipidemia, *n* (%)	3 (4.8) ^1^		7 (17.1)		0.037 ^2^
Ischemic heart disease, *n* (%)	2 (3.8) ^1^		3 (7.3)		0.350 ^2^

^1^ The value represents *n* (%), ^2^ chi square test; ^3^ *t*-Student test; ^4^ Mann–Whitney test; abbreviations: IQRs—quartiles; Ds—diopters; SD—standard deviation.

**Table 2:** Comparison of demographic, ocular, and systemic characteristics between the Alpha group and the control group, as well as between the Delta group and the control group.

Variables	COVID-19 Patients Alpha Group		COVID-19 Patients Delta Group		Control Group		*p* (Alpha vs. Control Group)	*p* (Delta vs. Control Group)
	Mean (SD)	Median (IQR)	Mean (SD)	Median (IQR)	Mean (SD)	Median (IQR)		
Men, *n* (%)	43 (68.3) ^1^		17 (58.5) ^1^		27 (61.4) ^1^		0.585 ^2^	0.194 ^2^
Women, *n* (%)	20 (31.7) ^1^		24 (41.5) ^1^		17 (38.6) ^1^			
Age (years)	51.3 (11.5)	51.0 (18.0)	53.71 (11.9)	54.0 (14.0)	47.8 (9.3)	47.0 (10.0)	0.087 ^3^	0.011 ^3^
Body height (cm)	173.1 (9.5)	176.0 (15.0)	170.5 (8.6)	172.0 (14.0)	171.2 (7.2)	170.0 (12.0)	0.214 ^3^	0.775 ^3^
Body mass (kg)	85.4 (15.6)	88.0 (20.0)	77.5 (19.8)	75.0 (16.0)	78.4 (15.6)	78.0 (23.0)	0.015 ^4^	0.411 ^4^
BMI (kg/m^2^)	28.4 (4.1)	28.0 (6.0)	26.5 (5.4)	25.28 (3.5)	26.8 (4.3)	26.5 (6.0)	0.026 ^4^	0.289 ^4^
LogMar Visual acuity	0.3 (0.04)	0.3 (0.0)	0.3 (0.0)	0.30 (0.0)	0.0 (0.0)	0.0 (0.0)	0.149 ^4^	0.999 ^4^
LogMar Reading vision	0.00 (0.02)	0.5 (0.0)	0.0 (0.0)	0.00 (0.0)	0.3 (0.0)	0.3 (0.0)	0.241 ^4^	0.999 ^4^
Intraocular pressure mmHg	16.4 (2.6)	16.5 (3.8)	16.2 (3.0)	16.34 (5.4)	16.4 (3.1)	16.4 (4.5)	0.921 ^3^	0.684 ^3^
Spherical equivalent (D)	0.13 (1.5)	0.0 (2.3)	−0.06 (1.4)	0.0 (1.5)	−0.7 (1.1)	−0.6 (1.4)	<0.001 ^4^	0.002 ^4^
Axial length (mm)	23.6 (0.9)	23.5 (1.1)	23.6 (0.9)	23.5 (1.0)	23.3 (0.9)	23.5 (1.2)	0.119 ^4^	0.131 ^4^

^1^ The value represents *n* (%), ^2^ chi square test; ^3^ *t*-Student test; ^4^ Mann–Whitney test; abbreviations: IQRs—quartiles; Ds—diopters; SD—standard deviation.

**Table 3:** Baseline laboratory parameters in two studied COVID-19 groups, Alpha and Delta, and reference values.

Parameters	COVID-19 Patients Alpha Group		COVID-19 Patients Delta Group		References Values	*p*
	Mean (SD)	Median (IQR)	Mean (SD)	Median (IQR)		
Saturation (%)	93.3 (3.95)	94.0 (92.0–96.0)	93.6 (3.68)	95.0 (91.0–96.0)	95–99	0.854 ^2^
CRP (mg/L)	64.0 (64.2)	37.0 (13.9–96.1)	57.4 (68.88)	28.6 (11.5–93.2)	<1.0	0.594 ^2^
Procalcitonin (ng/mL)	0.17 (0.24)	0.08 (0.04–0.18)	0.09 (0.06)	0.07 (0.04–0.11)	0.05–0.1	0.332 ^2^
WBC (1/µL)	5723.2 (2312.7)	5200.0 (4300.0–6440.0)	4960.5 (1891.2)	4790.0 (3630.0–6100.0)	4000–10,000	0.116 ^2^
Lymphocytes (1/µL)	1222.3 (457.1)	1185.0 (910.0–1480.0)	1129.3 (470.6)	1000.0 (800.0–1350.0)	1000–5000	0.320 ^1^
Neutrophils (1/µL)	3997.3 (2111.2)	3550.0 (2500.0–4700.0)	3261.7 (1628.8)	3300.0 (2000.0–4000.0)	1800–8000	0.088 ^2^
PLT (1/µL)	205,411.3 (76,851.4)	191,500.0 (150,000.0–231,000.0)	211,048.8 (70,384.6)	208,000.0 (163,000.0–238.00)	150,000–400,000	0.454 ^2^
IL-6 (pg/mL)	35.8 (31.0)	28.03 (13.0–48.1)	34.9 (38.40)	14.39 (7.9–54.8)	<1.8	0.225 ^2^
D-dimers (ng/mL)	1095.3 (3297.8)	542.5 (381.0–701.0)	2465.5 (9530.5)	690.0 (550.0–1013.0)	<500	0.005 ^2^
ALT (IU/L)	56.5 (39.4)	46.50 (26.0–74.0)	47.44 (39.0)	35.0 (23.0–57.0)	5–40	0.093 ^2^

^1^ *t*-Student test; ^2^ Mann–Whitney test. Abbreviations: ALT—alanine aminotransferase; CRP—C reactive protein; WBC—white blood cells; IL-6—interleukin-6-; PLTs—platelets; IQRs—quartiles; Ds—diopters; SD—standard deviation.

**Table 4:** Systemic treatment due to SARS-CoV-2 infection in two COVID-19 study groups: Alpha and Delta.

Treatment	COVID-19 Patients Alpha Group	COVID-19 Patients Delta Group	*p*
Low-molecular-weight heparin—prophylactic dose, *n* (%)	59 (93.7)	38 (92.7)	0.847 ^1^
Low-molecular-weight heparin—prophylactic dose—number of days: median (IQR); min-max	9.0 (5.8); 2–30 days	9.0 (3.8); 1–19 days	0.653 ^2^
Low-molecular-weight heparin therapeutic dose *n* (%)	2 (3.2)	3 (7.3)	0.354 ^1^
Tocilizumab, *n* (%)	3 (4.9)	2 (4.9)	0.978 ^1^
Dexamethasone, *n* (%)	22 (34.9)	14 (34.2)	0.935 ^1^
Remdesiwir, *n* (%)	26 (41.3)	5 (12.2)	0.002 ^1^
Need for continuous oxygen therapy, *n* (%)	23 (36.5)	21 (51.2)	0.138 ^1^
Need for continuous oxygen therapy, number of days: median (IQR); min-max	5 (6); 2–26 days	6 (4); 2–16 days	0.879 ^2^

^1^ chi square test; ^2^ Mann–Whitney test; abbreviations: IQR—interquartile range.

**Table 5:** Comparison of OCTA parameters of the central retina and central choroid between two COVID-19 study groups, Alpha and Delta.

Variables	COVID-19 Patients Alpha Group		COVID-19 Patients Delta Group		*p*
	Mean (SD)	Median (IQR)	Mean (SD)	Median (IQR)	
VD F SCP, (μm)	20.7 (4.2)	20.9 (5.2)	21.2 (5.2)	21.4 (6.1)	0.534 ^1^
VD F DCP, (μm)	17.2 (4.7)	16.9 (6.4)	16.2 (6.2)	15.8 (6.2)	0.076 ^1^
VD F CCP, (μm)	51.4 (4.2)	52.1 (5.3)	53.1 (3.9)	53.4 (5.6)	0.005 ^1^
VD S SCP, (μm)	48.3 (2.7)	48.4 (2.7)	45.2 (2.6)	45.3 (2.9)	<0.001 ^1^
VD S DCP, (μm)	52.0 (3.3)	52.1 (4.5)	47.3 (2.7)	47.5 (2.7)	<0.001 ^2^
VD S CCP, (μm)	54.1 (2.0)	54.2 (2.4)	51.5 (2.1)	51.5 (2.7)	<0.001 ^1^
VD N SCP, (μm)	45.1 (2.6)	45.2 (3.6)	44.2 (2.7)	44.9 (3.3)	0.039 ^1^
VD N DCP, (μm)	49.0 (2.0)	48.6 (3.9)	45.4 (2.1)	45.7 (2.1)	<0.001 ^1^
VD N CCP, (μm)	53.6 (2.1)	53.6 (2.3)	52.3 (2.0)	52.5 (2.8)	<0.001 ^2^
VD I SCP, (μm)	47.2 (4.4)	47.7 (3.7)	44.8 (3.7)	45.2 (4.9)	<0.001 ^1^
VD I DCP, (μm)	51.3 (4.0)	51.1 (5.3)	44.5 (3.7)	44.8 (3.4)	<0.001 ^1^
VD I CCP, (μm)	54.3 (2.2)	54.3 (3.0)	51.9 (2.4)	51.8 (3.3)	<0.001 ^2^
VD T SCP, (μm)	46.7 (2.2)	46.6 (2.8)	45.1 (2.1)	45.1 (3.0)	<0.001 ^2^
VD T DCP, (μm)	47.6 (2.5)	47.8 (3.3)	43.5 (2.1)	43.3 (2.9)	<0.001 ^2^
VD T CCP, (μm)	54.0 (1.9)	53.9 (2.6)	53.2 (1.9)	53.2 (2.7)	0.002 ^1^
Mean VD SCP, (μm)	52.0 (2.4)	52.4 (2.8)	44.8 (2.3)	45.0 (3.2)	<0.001 ^1^
Mean VD DCP, (μm)	54.3 (2.6)	54.0 (3.8)	45.2 (1.6)	45.1 (2.4)	<0.001 ^2^
Mean VD CC, (μm)	66.9 (1.3)	66.9 (1.5)	52.2 (1.0)	52.3 (1.2)	<0.001 ^1^
FAZs (µm^2^)	326.8 (107.0)	323.9 (126.5)	282.1 (123.6)	255.4 (183.6)	0.002 ^1^
FAZd (µm^2^)	357.8 (144.3)	344.8 (169.3)	270.0 (105.9)	269.9 (145.0)	0.002 ^1^

^1^ Mann–Whitney test; ^2^ *t*-Student test; abbreviations: F—foveal; I—inferior; N—nasal; S—superior; T—temporal; CCP—choriocapillaris capillary plexus; DCP—deep capillary plexus; FAZd—foveal avascular zone deep; FAZs—foveal avascular zone superficial; IQR—interquartile range; OCTA—optical coherence tomography angiography; SCP—superficial capillary plexus; SD—standard deviation.

**Table 6:** Correlations (Spearman’s correlation coefficient, Rs) between OCTA parameters and saturation, interleukin-6, and d-dimer levels in patients from the Alpha and Delta groups.

	Saturation		IL-6		D-Dimer	
	Alpha	Delta	Alpha	Delta	Alpha	Delta
Mean VD SCP	0.01 (*p* = 0.87)	−0.06 (*p* = 0.60)	−0.02 (*p* = 0.82)	−0.01 (*p* = 0.97)	−0.10 (*p* = 0.28)	0.09 (*p* = 0.43)
Mean VD DCP	−0.05 (*p* = 0.58)	0.02 (*p* = 0.88)	0.03 (*p* = 0.72)	−0.11 (*p* = 0.34)	−0.11 (*p* = 0.25)	−0.05 (*p* = 0.65)
Mean VD CCP	−0.15 (*p* = 0.11)	0.10 (*p* = 0.39)	0.06 (*p* = 0.12)	−0.03 (*p* = 0.82)	0.15 (*p* = 0.10)	−0.18 (*p* = 0.13)

CCP—choriocapillaris capillary plexus; DCP—deep capillary plexus; IL-6—interleukin-6; SCP—superficial capillary plexus; VD—vessel density.

**Table 7:** Comparison of OCTA parameters of the central retina and central choroid between Alpha COVID-19 patients and control group.

Variables	COVID-19 Patients Alpha Group		Control Group		*p*
	Mean (SD)	Median (IQR)	Mean (SD)	Median (IQR)	
VD F SCP, (μm)	20.7 (4.2)	20.9 (5.2)	20.7 (4.06)	21.1 (5.3)	0.871 ^1^
VD F DCP, (μm)	17.2 (4.7)	16.9 (6.4)	16.9 (4.48)	16.5 (6.9)	0.849 ^1^
VD F CCP, (μm)	51.4 (4.2)	52.1 (5.3)	52.4 (4.30)	48.2 (3.8)	0.046 ^1^
VD S SCP, (μm)	48.3 (2.7)	48.4 (2.7)	48.2 (3.3)	48.2 (3.8)	0.708 ^1^
VD S DCP, (μm)	52.0 (3.3)	52.1 (4.5)	52.2 (3.6)	51.9 (4.6)	0.688 ^2^
VD S CCP, (μm)	54.1 (2.0)	54.2 (2.4)	54.2 (2.5)	54.2 (2.4)	0.422 ^1^
VD N SCP, (μm)	45.1 (2.6)	45.3 (3.6)	45.0 (2.4)	45.1 (3.6)	0.589 ^1^
VD N DCP, (μm)	49.0 (3.0)	48.6 (3.9)	48.5 (2.9)	47.9 (4.2)	0.160 ^2^
VD N CCP, (μm)	53.6 (2.1)	53.6 (2.3)	53.2 (2.4)	53.3 (2.9)	0.361 ^1^
VD I SCP, (μm)	47.2 (4.4)	47.7 (3.7)	47.0 (3.4)	47.5 (5.1)	0.840 ^1^
VD I DCP, (μm)	51.3 (4.0)	51.1 (5.3)	50.9 (3.6)	50.3 (4.4)	0.204 ^1^
VD I CCP, (μm)	54.3 (2.2)	54.3 (3.0)	54.3 (2.5)	54.1 (2.5)	0.739 ^1^
VD T SCP, (μm)	46.7 (2.2)	46.6 (2.8)	46.4 (2.2)	46.5 (3.3)	0.490 ^2^
VD T DCP, (μm)	47.6 (2.5)	47.8 (3.3)	47.2 (3.0)	46.9 (4.4)	0.293 ^2^
VD T CCP, (μm)	54.0 (1.9)	53.9 (2.6)	53.5 (4.5)	54.1 (2.4)	0.967 ^1^
Mean VD SCP, (μm)	52.0 (2.4)	52.4 (2.8)	51.8 (2.5)	51.9 (3.4)	0.745 ^2^
Mean VD DCP, (μm)	54.3 (2.6)	54.0 (3.8)	53.9 (2.4)	54.0 (3.9)	0.241 ^2^
Mean VD CC, (μm)	66.9 (1.3)	66.9 (1.5)	66.9 (1.8)	67.0 (2.0)	0.456 ^1^
FAZs (µm^2^)	326.8 (107.0)	323.9 (126.5)	251.0 (111.2)	243.1 (154.0)	<0.001 ^2^
FAZd (µm^2^)	357.8 (144.3)	344.6 (169.3)	235.1 (110.3)	226.4 (157.0)	<0.001 ^1^

^1^ Mann–Whitney test, ^2^ *t*-Student test; abbreviations: F—foveal; I—inferior; N—nasal; S—superior; T—temporal; CCP—choriocapillaris capillary plexus; DCP—deep capillary plexus; FAZd—foveal avascular zone deep; FAZs—foveal avascular zone superficial; IQR—interquartile range; OCTA—optical coherence tomography angiography; SCP—superficial capillary plexus; SD—standard deviation; VD—vessel density.

**Table 8:** Comparison of OCTA parameters of the central retina and central choroid between Delta COVID-19 patients and control group.

Variables	COVID-19 Patients Delta Group		Control Group		*p*
	Mean (SD)	Median (IQR)	Mean (SD)	Median (IQR)	
VD F SCP, (μm)	21.2 (5.2)	21.4 (6.1)	20.7 (4.1)	21.2 (5.3)	0.628 ^2^
VD F DCP, (μm)	16.2 (6.2)	15.8 (6.2)	15.8 (6.3)	16.5 (6.9)	0.141 ^2^
VD F CCP, (μm)	53.1 (3.9)	53.4 (5.6)	53.4 (5.6)	48.2 (3.8)	0.416 ^2^
VD S SCP, (μm)	45.2 (2.6)	45.3 (2.9)	48.2 (3.4)	48.2 (3.8)	<0.001
VD S DCP, (μm)	47.3 (2.7)	47.5 (2.7)	47.5 (2.7)	51.9 (4.6)	<0.001 ^1^
VD S CCP, (μm)	51.45 (2.1)	51.5 (2.7)	51.5 (2.7)	54.2 (2.4)	<0.001 ^2^
VD N SCP, (μm)	44.2 (2.7)	44.9 (3.3)	45.0 (2.4)	45.1 (3.6)	0.060 ^1^
VD N DCP, (μm)	45.4 (2.1)	45.7 (2.1)	45.7 (2.1)	47.9 (4.2)	<0.001 ^2^
VD N CCP, (μm)	52.3 (2.0)	52.7 (2.8)	52.5 (2.8)	53.3 (2.9)	<0.005 ^2^
VD I SCP, (μm)	44.8 (3.7)	45.2 (4.9)	47.0 (3.3)	47.5 (5.1)	<0.001 ^2^
VD I DCP, (μm)	44.5 (3.7)	44.8 (3.4)	44.8 (3.4)	50.3 (4.3)	<0.001 ^2^
VD I CCP, (μm)	51.9 (2.4)	51.8 (3.3)	51.8 (3.3)	54.1 (2.5)	<0.001 ^2^
VD T SCP, (μm)	45.1 (2.1)	45.1 (3.1)	46.4 (2.3)	46.5 (3.3)	<0.001 ^2^
VD T DCP, (μm)	43.5 (2.1)	43.3 (2.9)	43.3 (2.9)	46.9 (4.4)	<0.001 ^1^
VD T CCP, (μm)	53.2 (1.9)	53.2 (2.7)	53.2 (2.7)	54.1 (2.4)	0.005 ^2^
Mean VD SCP, (μm)	44.8 (2.3)	45.0 (3.2)	45.0 (3.2)	51.8 (3.4)	<0.001 ^2^
Mean VD DCP, (μm)	45.2 (1.6)	45.1 (2.4)	45.1 (2.4)	54.0 (3.9)	<0.001 ^1^
Mean VD CC, (μm)	52.2 (1.0)	52.3 (1.2)	52.23 (1.2)	67.0 (2.0)	<0.001 ^2^
FAZs (µm^2^)	282.1(123.6)	255.4 (183.6)	255.4 (183.6)	243.1 (154.0)	0.002 ^2^
FAZd (µm^2^)	270 (105.9)	269.9 (145.0)	269.9 (145.0)	226.4 (157.0)	0.002 ^2^

^1^ *t*-Student test; ^2^ Mann–Whitney test; abbreviations: F—foveal; I—inferior; N—nasal; S—superior; T—temporal; CCP—choriocapillaris capillary plexus; DCP—deep capillary plexus; FAZd—foveal avascular zone deep; FAZs—foveal avascular zone superficial; IQR—interquartile range; OCTA—optical coherence tomography angiography; SCP—superficial capillary plexus; SD—standard deviation; VD—vessel density.

## Data Availability

The data supporting the reported results can be provided upon request from the corresponding author.
